# Major risk factors and histopathological profile of treatment failure, relapse and chronic patients with anthroponotic cutaneous leishmaniasis: A prospective case-control study on treatment outcome and their medical importance

**DOI:** 10.1371/journal.pntd.0009089

**Published:** 2021-01-28

**Authors:** Mehdi Bamorovat, Iraj Sharifi, Shahriar Dabiri, Simin Shamsi Meymandi, Ali Karamoozian, Rezvan Amiri, Amireh Heshmatkhah, Mehdi Borhani Zarandi, Mohammad Reza Aflatoonian, Fatemeh Sharifi, Reza Kheirandish, Saeid Hassanzadeh

**Affiliations:** 1 Leishmaniasis Research Center, Kerman Univeprsity of Medical Sciences, Kerman, Iran; 2 Department of Pathology, Afzalipour Hospital, Pathology and Stem Cell Research Center, Kerman University of Medical Sciences, Kerman, Iran; 3 Department of Dermatology, Pathology and Stem Cell Research Center, Kerman University of Medical Sciences, Kerman, Iran; 4 Research Center for Modeling in Health, Institute for Futures Studies in Health, Kerman University of Medical Sciences, Kerman, Iran; 5 Department of Dermatology, Afzalipour Hospital, Kerman University of Medical Sciences, Kerman, Iran; 6 Dadbin Health Clinic, Kerman University of Medical Sciences, Kerman, Iran; 7 Research Center for Hydatid Disease in Iran, Kerman University of Medical Sciences, Kerman, Iran; 8 Research Center for Tropical and Infectious Diseases, Kerman University of Medical Sciences, Kerman, Iran; 9 Pharmaceutics Research Center, Institute of Neuropharmacology, Kerman University of Medical Sciences, Kerman, Iran; 10 Department of Pathobiology, Faculty of Veterinary Medicine, Shahid Bahonar University of Kerman, Kerman, Iran; Federal University of Bahia, BRAZIL

## Abstract

Over the last years, there has been a remarkable increase in the number of unresponsive patients with anthroponotic cutaneous leishmaniasis (ACL) reported worldwide. The primary objective of this study was to explore the role of demographic, clinical and environmental risk related-factors in the development of treatment failure, relapse and chronic cases compared to responsive patients with ACL. Moreover, molecular, histopathological and immunohistochemical (IHC) findings between these forms were explored. This work was undertaken as a prospective and case-control study in southeastern Iran. Culture media and nested PCR were used to identify the causative agent. Univariate multinomial and multiple multinomial logistic regression models and the backward elimination stepwise method were applied to analyze the data. A *P*<0.05 was defined as significant. Also, for different groups, skin punch biopsies were used to study the histopathological and immunohistochemical (IHC) profile. All samples showed that *L*. *tropica* was the only etiological agent in all unresponsive and responsive patients with ACL. Data analysis represented that 8 major risk factors including nationality, age groups, occupation, marital status, history of chronic diseases, duration of the lesion, the lesion on face and presence of domestic animals in the house were significantly associated with the induction of unresponsive forms. The histopathological and immunohistochemical findings were different from one form to another. The present findings clearly demonstrated a positive relation between ACL and distinct demographic, clinical and environmental risk determinants. Knowledge of the main risk factors for ACL infection is crucial in improving clinical and public health strategies and monitor such perplexing factors.

## Author summary

Negligible data are present related to anthroponotic cutaneous leishmaniasis (ACL) treatment outcome and resultant unresponsiveness risk determinants. The role of demographic, clinical, and environmental risk associated-factors in the development of treatment failure, relapse, and chronic forms of ACL has not been studied. We carried out a case-control study for a period of 4 years (2015–2019) using culture media and nested PCR to identify the causative agent. Afterward, we analyzed the data by univariate multinomial and multiple multinomial logistic regression models and the backward elimination stepwise method. Also, we examined skin punch biopsies to study the histopathological and immunohistochemical (IHC) profile for different comparative groups. The findings identified 8 major risk factors were significantly associated with the creation of unresponsive forms. Clinical practitioners and health surveillance systems should be aware of and monitor such perplexing factors. Awareness of the major determinants for unresponsiveness to the treatment of ACL is critical to improving clinical strategies and public health measures. These multidisciplinary approaches need to address specific barriers that directly affect the treatment outcome.

## Introduction

Leishmaniasis comprises a range of complex diseases with diverse clinical features caused by the genus *Leishmania*. The disease consists of three unique groups of the syndrome including cutaneous leishmaniasis (CL), visceral leishmaniasis (VL) and mucocutaneous leishmaniasis (MCL) [1–3]. Currently, more than 1 billion inhabitants in over 100 countries have been at risk of infection[4] and CL is the most prevalent form of the disease[1, 5]. This disease has recently become one of the major global health problems around the world especially in the Eastern Mediterranean Region [6].

With high outbreak rates, anthroponotic CL (ACL) caused by *Leishmania* (*L*.) *tropica* and zoonotic CL (ZCL) caused by *L*. *major* is endemic in several parts of Iran and also ACL has been present for centuries in the country[7–9]. In urban areas, CL is usually due to *L*. *tropica* which is transmitted from human-to-human by the female sand fly bites, *Phlebotomus (Ph*.*) sergenti* (anthroponotic cycle) [10]; although, dogs have been found to be sporadically infected[11]. According to the recent reports carried out in the southeast of Iran, all examined CL patients (unresponsive and responsive to meglumine antimoniate) were infected by *L*. *tropica* species[12–15].

Leishmaniasis is basically endemic in poverty-ridden locations and some of the clinical cases are not treated properly due to the lack of active case detection by the public health surveillance system [16, 17].

The disease is directly linked to multiple determinants such as clinical, socioeconomic, cultural, religious, demographic, and environmental contributing factors. Movement of manual laborers and migrants from rural to urban areas for work-related reasons and conflicts can be the most important risk factors for ACL[5, 13, 18]. The geographical distribution of leishmaniasis is dynamic and is reported to (re-) emerge and expand its original territories to new areas in many countries [18, 19]. Control of the disease is challenging due to three main risk factors: anthropogenic/environmental changes, host’s immune responses, and treatment failure to conventional drugs [13, 18–20].

Due to the complexity of the life cycle, unavailability of the human vaccine and the presence of so many biological vectors and reservoir hosts, the control of the disease remains unresolved. Therefore, chemotherapy is still the main strategy to control the disease [21]. Meglumine antimoniate (MA; Glucantime) is the gold standard treatment for CL in Iran. The MA resistant *L*. *tropic* parasites have appeared from unresponsive cases in the form of chronic, relapse and treatment failure. There are also diverse factors that affect the unresponsiveness to the treatment of leishmaniasis such as social and geographic grounds, clinical characteristics and type of diseases, *Leishmania* species and host’s immune response [13, 21–25]. Recently, challenges in the chemotherapy of leishmaniasis have caused a considerable resistance to MA [3, 23]; although, unresponsiveness to treatment is a complex phenomenon with potentially multifactorial causes.

The primary objective of the present study was to explore the role of demographic, clinical and environmental risk related-factors in the development of treatment failure, relapse and chronic cases compared to responsive patients with ACL. Furthermore, the comparison of histopathological and immunohistochemical (IHC) findings between these forms has been explored.

## Methods

### Ethics statement

Ethical considerations were approved by the joint Ethics Committees of the Kerman University of Medical Sciences and Kerman Leishmaniasis Research Center (ethics no. IR.KMU.REC.1398.083, contract no. 97001076). Initially, many face-to-face meetings and interviews with the patients and community health authorities were organized. The primary aim of the study and the potential benefits of the project were explained. A written informed consent form was completed for each patient. CL patients participated voluntarily in the study. All data were retained confidential. Moreover, “on behalf" of all the children, parents/guardians completed the written informed consent. All patients were treated with proper drugs without any charge. History of unresponsive patients who had already been recorded in the electronic file and also in a backup registration book in the referral clinic was used as the source of data for demographical and clinical characteristics.

### Design and type of study

This work was undertaken as a prospective and case-control study between January 2015 and June 2019 from high-risk zones of the district of Kerman in southeastern Iran. The present study consisted of four comparable arms of relapse, treatment failure and chronic patients versus the responsive control group.

### Study site and data collection

Kerman is the largest province in Iran is located in the southeast and about 1,000 km distant from Tehran. This province falls into the hot and dry zones and suffers from the shortage of water, conditions similar to much of the Iranian plateau. The regular yearly rainfall is low and maximum precipitation happens in winter; the annual average is 140–150 mm in Kerman. The district of Kerman is one of the well-known foci of ACL in Iran[9]. The present study was performed at Dadbin Health Center, the main referral clinic for CL treatment and control activities. The clinic is directly linked with the Kerman Leishmaniasis Research Center and School of Medicine in Kerman province. The clinic is in charge of the patients with CL who referred from different localities within the district. Every CL patient has a file recording demographic and clinical status. The structured form of the study was designed according to the CL indicators. In fact, some of the queries of the data collection form were chosen based on the previous cohorts and the leishmaniasis expert group[13, 26, 27]. Furthermore, the content was assessed by investigators from different fields including dermatology, parasitology and epidemiology in terms of its accuracy. Essential details about the study were mentioned to the volunteer patients or their guardians. Throughout the course of interviewing, the evaluators made sure that the participants or their guardians are well-understood the queries. Additionally, data were collected by house-to-house visits. Also, direct visit of housing and environmental hygienic conditions by the interviewers was documented with the consent of the households' heads. The questionnaire included demographic, clinical and environmental risk-related factors. The sample size was selected according to the rate of responsive and unresponsive patients in this region (based on our previous studies, knowledge and experience of the ACL cases referred to the registry center in Kerman), which in this regards, unresponsive patients are significantly lower than the control group (responsive patients). Some patients whose names, demographics, and clinical characteristics were recorded in the registry system but were absent at the house during the follow-up assessments unable to record environmental data and were excluded from the study. Patients were selected in a major ACL focus from treatment failure, relapse, chronic and responsive forms as case and control groups. Overall, n = 338 cases out of n = 392 ACL patients consisting of various clinical forms (n = 22 relapse, n = 38 treatment failure, n = 21 chronic, and n = 257 responsive cases) were selected and evaluated.

### Case-definition

**Fig 1:**
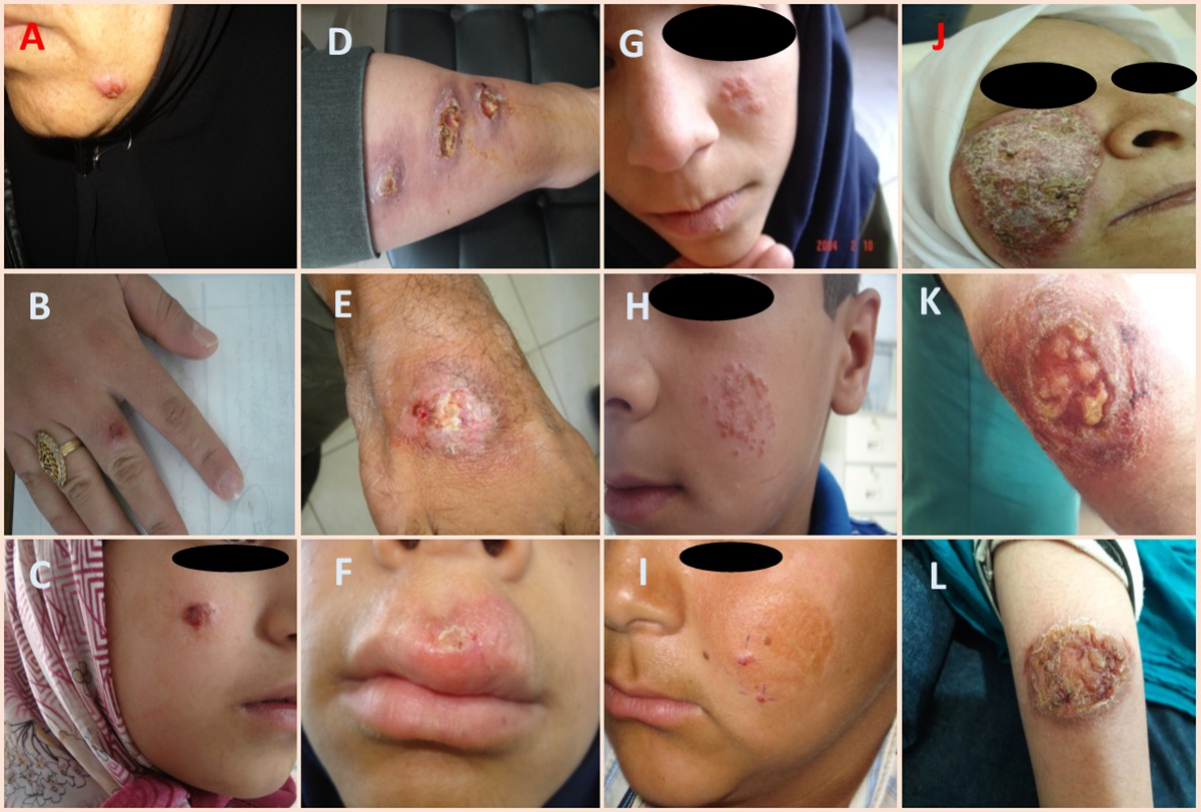
Representative images of various clinical forms of anthroponotic cutaneous leishmaniasis from Kerman district. A) a responsive form with erythematous nodular indurated plaque on the face, B) a responsive form with a crusted nodule on the hand, C) a responsive form with a crusted plaque on the face, D) a treatment failure form consisting of three ulcerated indurated erythematous plaques on the leg, E) a treatment failure form with an indurated crusted plaque on the leg, F) a treatment failure form with a crusted large plaque on the lip, G) a relapse (lupoid) form with erythematous apple jelly-like indurated papules around and within the scar on the cheek, H) a relapse (lupoid) form with apple jelly nodules around and within scar of the left cheek [21], I) a relapse (lupoid) form with popular lesion around the scar on the left cheek, J) a chronic form with a large erythematous crusted scaling indurated plaque on the right cheek, K) a chronic form with indurated erythematous exudation ulcer of the forearm, L) a chronic form with an ulcerated plaque of elbow area.

Patients were selected in a major ACL focus from treatment failure, relapse, chronic and responsive forms as case and control groups. During over 20 years the experiences, observations, and investigations of our team, which includes experts in medicine, parasitology, dermatology, histopathology, and epidemiology, four forms of ACL have been identified and proven in this region according to the following definitions. The responsive patient is the one whose skin lesion has healed by one complete course of treatment with intramuscular administration of MA or intra-lesional MA together with bi-weekly liquid nitrogen cryotherapy (complete re-epithelialization before day 45) (Table 1) as confirmed by a 6-month follow-up assessment to make sure that no relapse has been occurred (Fig 1A, 1B and 1C). A treatment failure patient is one who had not healed (an increase of a plaque, nodule or ulcer following treatment and incomplete re-epithelialization after 45 days of treatment commencement) and remains with an active cutaneous lesion, despite obtaining two complete courses of intra-lesional MA (20 mg/kg every week for 12 weeks) along with bi-weekly cryotherapy or systemic MA alone (20 mg/kg every day for 21 days) and then after receiving the third course of treatment whose lesion had healed (confirmed by a 6-month follow-up assessment to make sure that no relapse has occurred) (Fig 1D, 1E and 1F). Relapse (lupoid leishmaniasis) patient is one who had healed (clinical cure) and after months or years specific plaque, nodule or ulcer reappear around or in the old scar (Fig 1G, 1H and 1I). In contrast, cases who do not heal and remain with an active skin lesion despite receiving over three complete courses of intra-lesional MA along with bi-weekly cryotherapy or systemic MA and also at least two years from the appearance of the lesion have passed, considered as a chronic form (Fig 1J, 1K and 1L) [28, 29]. The patients for their demographic, clinical and environmental risk-related factors were compared.

**Table 1:** The information and criteria for assigning meglumine antimoniate route.

Type of treatment	Reasons of treatment type	Timing and duration of the treatment	Required dosage
^a^IM meglumine antimoniate (MA)	-the number of lesions > 5—lesions ≥ 3 cm in diameter -the lesion close to vital organs	every day for 21 days	20 mg/kg of MA
^b^IL MA+ Cryotherapy	-the number of lesions < 5—lesions ≤ 3 cm in diameter -the lesions which are not close to vital organs	every week for 12 weeks along with bi-weekly cryotherapy	20 mg/kg of MA

^a^IM: intramuscular injection

^b^IL: intralesional injection.

### Culturing of the parasite

Culture media were used to identify the causative agent. Out of 40 patients in the study, for each group (relapse, treatment failure, chronic or responsive) 10 cases were randomly selected. The sampling method was carried out with an active skin lesion in four groups of patients. The lesion of patients was washed and cleansed with topical antiseptic and then it was aspirated by inserting a 26-gauge needle with a syringe. Parasites were cultured in Novy-MacNeal-Nicolle medium at 24±1°C for 7 days; after which, cultured in RPMI1640 medium (Gibco BRL) with 10% inactive fetal calf serum (Sigma-Aldrich), streptomycin (100 μg/ml) and penicillin (100 IU/ml)[27]. Parasites were obtained by 5 min centrifugation at 8000 rpm and the remaining samples were kept at -20°C for additional molecular examinations.

### Molecular characterization

DNA was extracted from all parasite samples using QIAamp DNA Minikit (Qiagen, Germany) based on the manufacturer's instructions. Species characterization of the *Leishmania* isolates was done using nested PCR of kinetoplast DNA (kDNA) by specific primers of CSB2XF (CGA GTA GCA GAA ACT CCC GTT CA) and CSB1XR (ATT TTT CGC GAT TTT CGC AGA ACG) as external primers and 13Z (ACT GGG GGT TGG TGT AAA ATA G) and LiR (TCG CAG AAC GCC CCT) as internal primers, which can specifically distinguish among several *Leishmania* species[30].

### Histopathological study

For each group, one case which had typical characteristics of the form was selected. The punch biopsies were taken from the lesion located in a responsive patient from a hand, in a treatment failure patient from a hand, a relapse patient from the face, and in a chronic patient from the face.

The clinical diagnosis was parasitologically confirmed by Giemsa staining of smear and also polymerase chain reaction (PCR) analysis. Skin biopsies were fixed in 10% formalin and then paraffin blocks were obtained. Afterward, sections with 4 μm thickness were prepared and hematoxylin and eosin (H&E) staining were performed. The histopathological parameters including parakeratosis, hyperkeratosis, acanthosis, ulceration, exocytosis, spongiosis, abscess formation, apoptotic body, melanophages collection, atrophy epidermis, pseudo-epitheliomatous hyperplasia, congestion and inflammatory cells infiltration in the dermis were studied according to previous CL histopathological studies[31].

### Immunohistochemical test (IHC)

Four μm thickness sections were provided. Sections were salinized to develop the adherence of tissue. Following dewaxing and rehydrating, with antigen retrieval unmasking antigens were done in 0.01 M citrate buffer into the microwave oven for 10 min at 800 W. Subsequently, sections were slowly cooled to room temperature. The next slides were washed rapidly in Tris-buffered saline at pH 7.4 and then stained with immunohistochemical markers including anti-CD68, anti-CD3, anti-CD1a, anti-CD20 and 3, 3’-diaminobenzidine (DAB) chromogen. These markers were monoclonal antibodies applied for immunophenotypic detection of leukocytes, directed to the following human cell surface antigens and included CD3 (code MU322-UC; clone PS1), CD20 (code MU238A-UC; clone L26), CD68 (code MU416-UC; clone KP1), CD1a (code MU490-UC; clone O10), and S100 (code NU713-UC; clone EP32) (BioGenex company), prepared to be used without dilution factor. EnVision solution was applied which contained secondary antibody bind to the biotin and streptavidin bind to peroxidase. Different inflammatory cells (cells/mm^2^) were counted using a light microscope, an ocular micrometer and a manual hematology cell counter. All required fields were counted in each section at a magnification of 400X[31].

### Data analysis

The data were entered into a computer and analyzed using a SPSS software (version 21.0, SPSS, Inc., Chicago, IL, USA). A *P*<0.05 was defined as significant. Univariate multinomial logistic regression was used to assess if the variables were suitable to be used in multiple multinomial logistic regressions. At last, variables with a *P*-value of less than 0.2 were picked after the analysis. The major reason for using the multiple methods was to exclude the confounders. To further control the confounders, the backward elimination stepwise method was applied to obtain the finest possible model. Concerning the rate of unresponsive cases (treatment failure, relapse, and chronic) in this region (10 to 12%), which is significantly lower than the control group (responsive patients), this sample size (n = 72) was designated.

## Results

### Molecular finding

**Fig 2:**
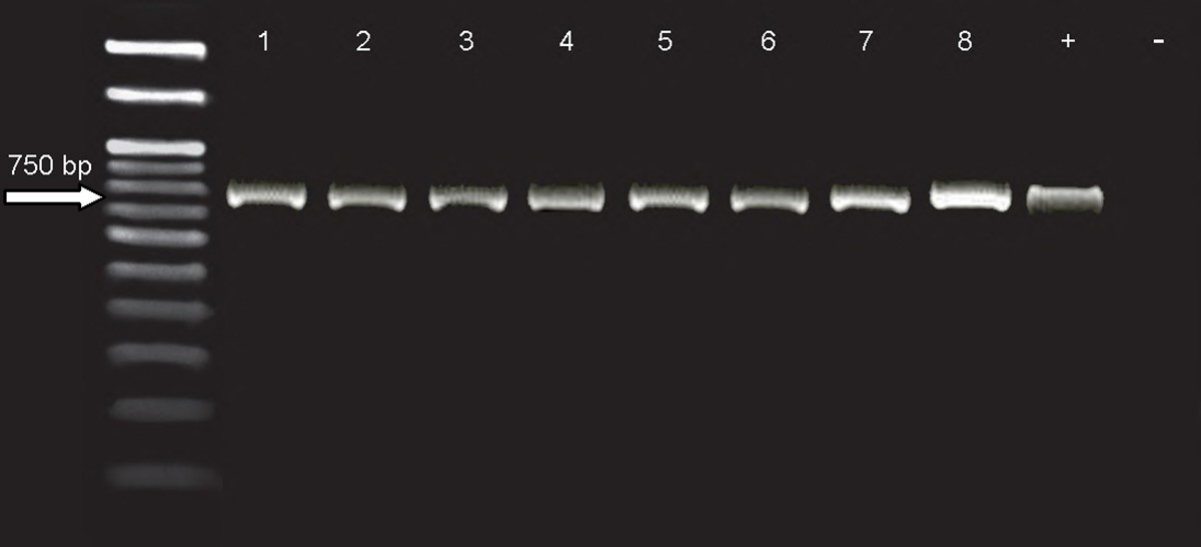
Representative agarose gel electrophoresis images. All *Leishmania* isolates from patients with anthroponotic cutaneous leishmaniasis areas of Kerman district were identified to be *L*. *tropica* species b*y* nested PCR (1 and 2: *Leishmania* isolates from responsive patients, 3 and 4: *Leishmania* isolates from treatment failure patients, 5 and 6: *Leishmania* isolates from relapse patients, 7 and 8: *Leishmania* isolates from chronic patients, standard *Leishmania tropica* and distilled water as positive and negative controls, respectively).

Exact detection and further approval of *Leishmania* isolates were based on positive cutaneous smear preparation, the culture of parasites and nested PCR methods. All samples showed a specific band of 750 bp with nested PCR, which displayed that *L*. *tropica* was the only species characterized as the etiological agent in all forms of ACL patients (Fig 2).

### Histopathological and immunohistochemical analyses

The following histopathological and immunohistochemical profiles were used as complementary data to confirm the outcomes.

Histopathological findings in four different forms of ACL were as follows.

**Fig 3:**
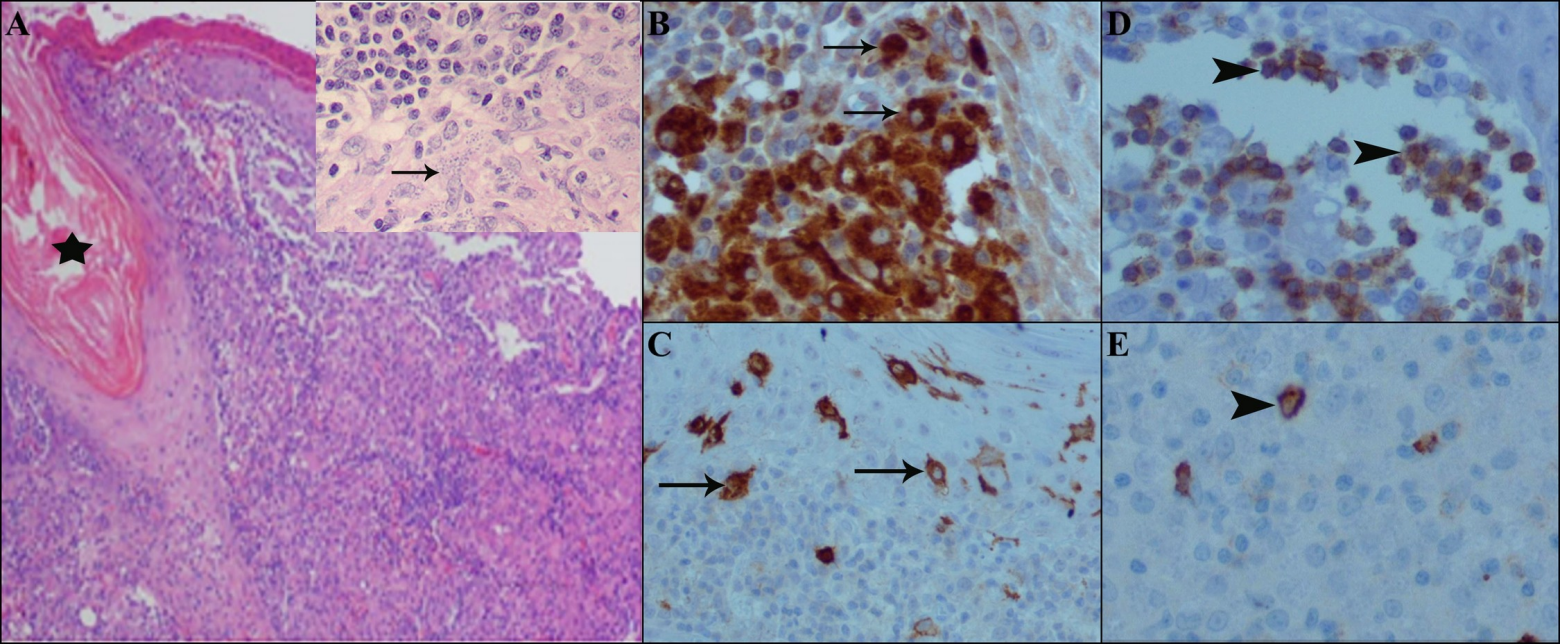
Histopathology and immunohistochemical findings in responsive form. A) H & E staining shows follicular plugging (star) and acanthosis, dermis displaying dense histiocytic and lymphocytic infiltrate (empty square and arrow) and many intracytoplasmic Leishman bodies, B) aggregation of histiocytes by CD68 IHC staining (arrows), C) collection of Langerhans cells in epidermis mostly, and dispersedly upper dermis by CD1a (arrows), D) CD3 lymphocytes infiltrate between histiocytes (arrowheads), E) rarity of positive B lymphocytes by CD20 IHC staining (arrowhead).

The responsive form (healed lesion) displayed a spectrum of epidermal changes such as ulceration, hyperkeratosis, parakeratosis, spongiosis, acanthosis and/or occasional inter epidermal migration of Leishman bodies. Dermis showed dense and diffuse proliferation mainly by histiocytes which were engulfed by intracytoplasmic Leishman bodies. They were entrapped by lymphocytes, partly plasma cells and eosinophils (Fig 3).

**Fig 4:**
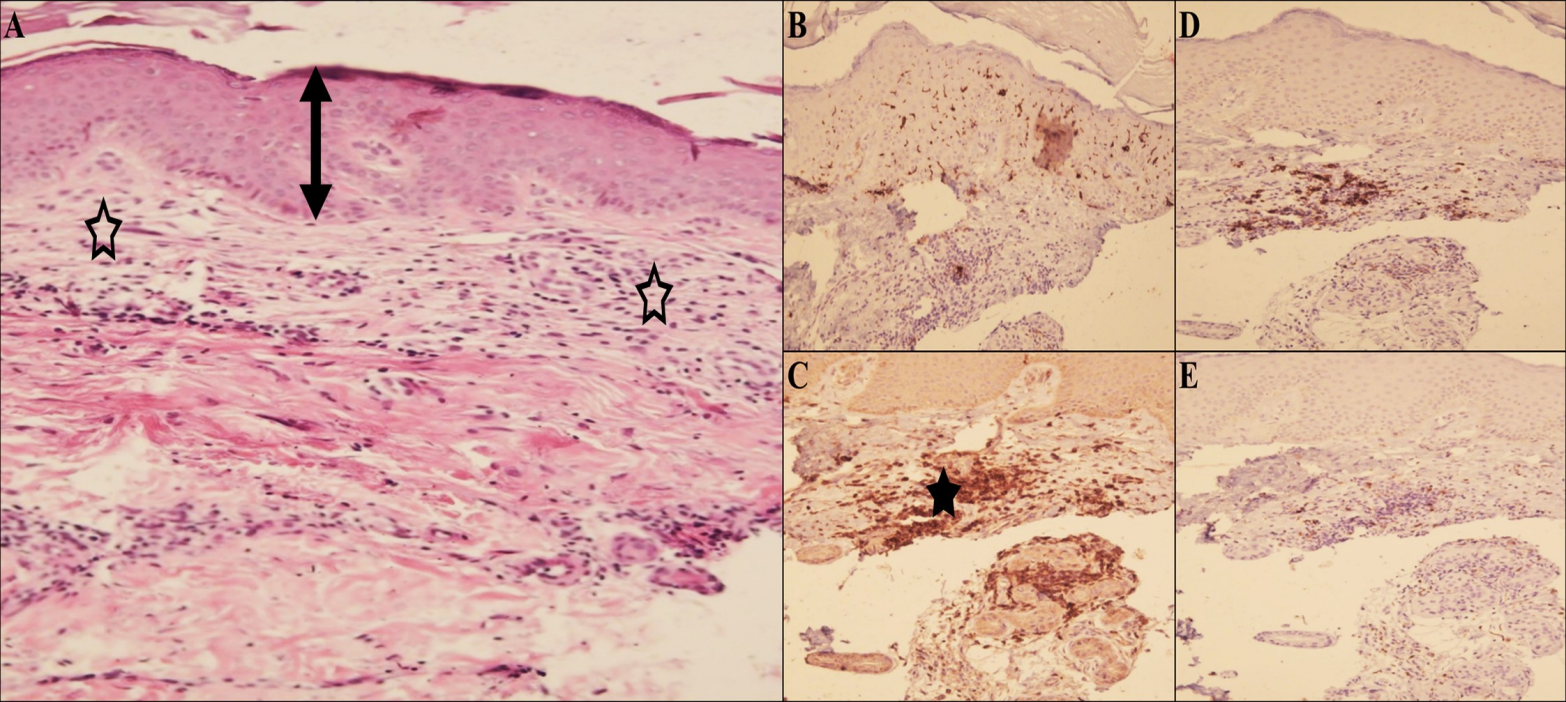
Histopathological and immunohistochemical findings in the treatment failure form. A) H & E staining shows histopathological changes of hyperkeratosis and acanthosis epidermis (double headed arrow) and dermis displaying histiocytic, lymphocytic and fibrohistiocytic cells infiltrate (empty stars), B) collection of Langerhans cells lattice in epidermis mostly, and dispersedly upper dermis by CD1a IHC staining, C) aggregation of histiocytes by CD68 IHC staining (star), D) lymphocytes infiltrate between histiocytes by CD3, E) rarity of positive B lymphocytes by CD20 IHC staining.

The treatment failure form demonstrated mild orthokeratosis and moderate acanthosis of the epidermis. Dermis showed a reduction in the number of macrophages and an increase in the density of lymphocytes and fibrohistiocytic cells. Leishman bodies were rarely found (Fig 4).

**Fig 5:**
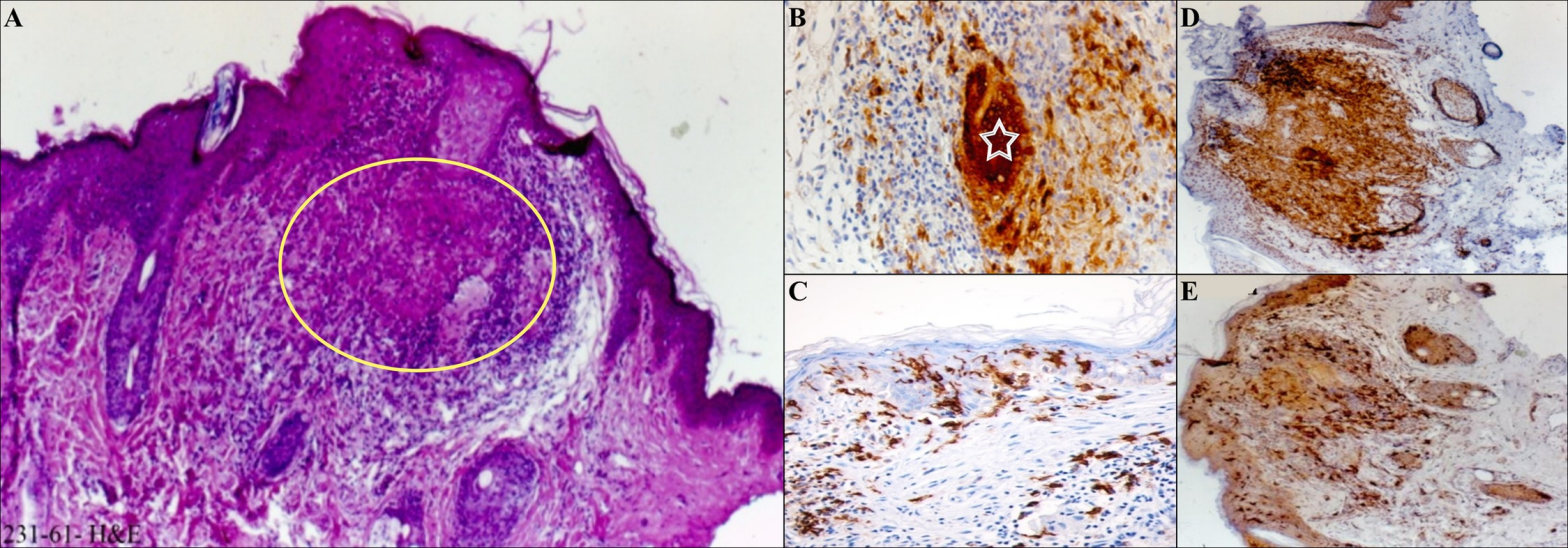
Histopathological and immunohistochemical findings in relapse (lupoid) form. A) H & E staining shows histopathological changes of thin epidermis and non-caseating epithelioid granuloma (empty circle) in the dermis, B) aggregation of histiocytes and Langhans giant cell by CD68 IHC staining (empty star), C) S100 and E) CD1a, network lattice like collection of Langerhans cells in epidermis and dermis and even in the granuloma, D) CD3, T-lymphocytes infiltrate between the histiocytes.

The relapse (lupoid) form exhibited atrophic to the focal acanthotic epidermis. Dermis showed multiple non-caseating epithelioid granuloma composed of epithelioid macrophages and Langerhans histiocytic giant cells. No Leishman bodies were found (Fig 5).

**Fig 6:**
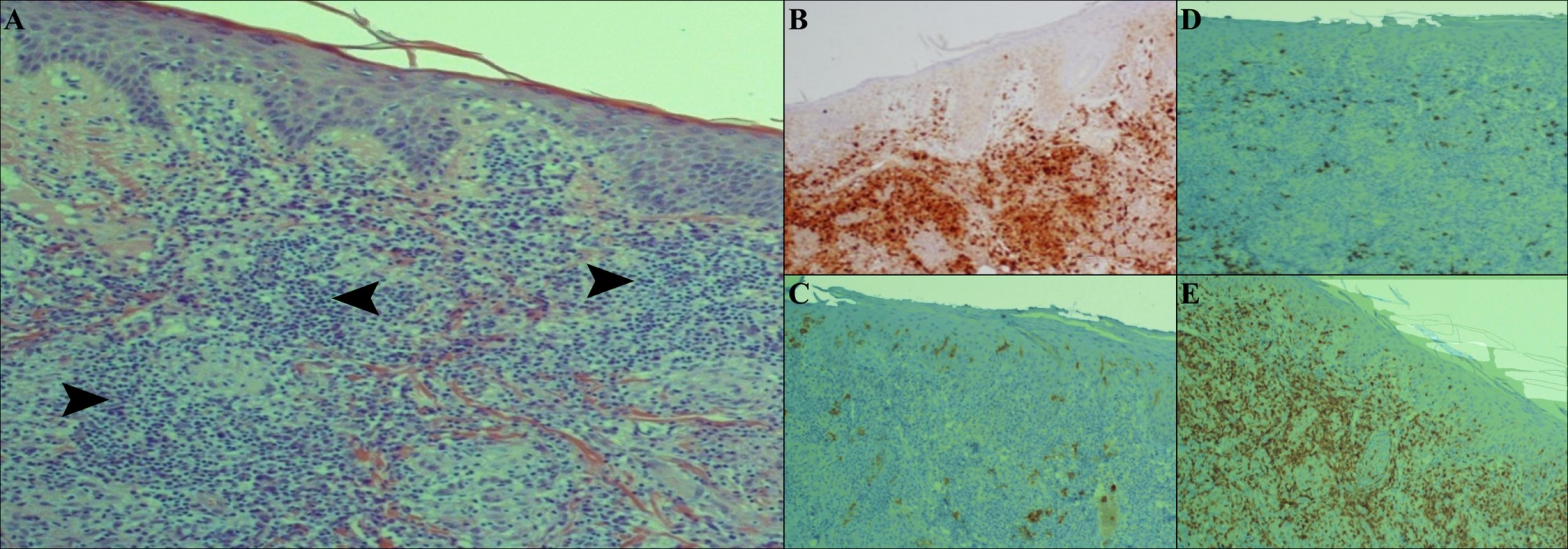
Histopathological and immunohistochemical findings in chronic form. A) H & E staining shows histopathological changes of dense and diffuse lymphohistiocytic infiltrate (arrowheads) in the dermis, B) aggregation of histiocytes by CD68 IHC staining, C) haphazard distribution of Langerhans cells in epidermis and dermis by CD1a IHC staining, D) presence of few infiltrate of positive B lymphocytes by CD 20 IHC staining, E) heavily infiltrate of CD3 T-lymphocytes between histiocytes.

The chronic form showed acanthosis and hyperkeratosis of the epidermis. Dermis represented diffuse multifocal or granulomatous collections of histiocytes intermixing with lymphocytes and occasional plasma cells. No Leishman bodies were visible in H&E stained section (Fig 6).

Also, our immunohistochemical findings by the CD68 marker on macrophages confirmed that positive cells were increased in number with a decrease of frequency in responsive, chronic, lupoid and treatment failure. CD1a was prominent in the responsive form in epidermis and dermis. But CD1a in lupoid and treatment failure was increased in the epidermis in order of frequency. CD3 was in between and rimming the histiocytic cells. CD20 was increased in chronic and treatment failure forms, but in responsive and lupoid forms were scattered.

### Risk factor analysis

Overall, 338 ACL patients including 22 (6.5%) relapse, 38 (11.2%) treatment failure, 21 (6.2%) chronic and 257 (76%) responsive patients with acute lesions or scars were precisely analyzed for possible demographical (Table 2), clinical (Table 3) and environmental factors (Table 4).

**Table 2:** Univariate and multiple multinomial logistic regression analyses for demographical risk related-factors: odds ratio of formation of chronic, treatment failure and relapse forms compared to responsive cases with anthroponotic cutaneous leishmaniasis in southeastern Iran.

Treatment outcome	Variables No. (%)	Univariate multinomial logistic regression			Multiple multinomial logistic regression		
		OR	CI (95%)	P-value	OR	CI (95%)	P-value
	**Nationality**						
Relapse	Afghani 2 (9.1)	0.88	(0.19–4.01)	0.87	1.02	(0.19–5.43)	0.97
	Iranian 20 (90.9)	1			1		
Treatment failure	Afghani 9 (23.7)	2.75	(1.17–6.45)	0.01	3.41	(1.11–10.43)	0.03
	Iranian 29 (76.3)	1			1		1
Chronic	Afghani 9 (42.9)	6.66	(2.56–17.31)	<0.001	8.10	(1.43–45.79)	0.01
	Iranian 12 (57.1)	1			1		
Responsive	Afghani 26 (10.1)						
	Iranian 231 (89.9)						
	**Age**						
Relapse	<8 8 (36.4)	1.01	(0.19–5.25)	0.98	0.77	(0.12–4.68)	0.77
	8–15 4 (18.2)	0.41	(0.06–2.43)	0.32	0.28	(0.04–1.93)	0.20
	16–30 3 (13.6)	0.46	(0.07–3.00)	0.41	0.37	(0.05–2.81)	0.34
	31–50 5 (22.7)	0.83	(0.14–4.72)	0.83	0.53	(0.08–3.46)	0.51
	>50 2 (9.1)	1			1		
Treatment failure	<8 6 (15.8)	0.21	(0.06–0.73)	0.01	0.19	(0.03–0.97)	0.04
	8–15 8 (21.1)	0.23	(0.07–0.73)	0.01	0.09	(0.01–0.44)	0.003
	16–30 8 (21.1)	0.35	(0.11–1.12)	0.07	0.23	(0.04–1.13)	0.07
	31–50 9 (23.7)	0.42	(0.13–1.33)	0.14	0.22	(0.04–1.08)	0.06
	>50 7 (18.4)	1			1		
Chronic	<8 7 (33.3)	1.77	(0.20–15.50)	0.60	0.41	(0.01–12.09)	0.61
	8–15 7 (33.3)	1.43	(0.16–12.49)	0.74	0.69	(0.02–17.70)	0.82
	16–30 3(14.3)	0.92	(0.09–9.50)	0.94	0.09	(0.002–3.22)	0.18
	31–50 3(14.3)	1.00	(0.09–10.37)	1.00	1.57	(0.05–49.59)	0.79
	>50 1(4.8)	1			1		
Responsive	<8 63(24.5)						
	8–15 78(30.4)						
	16–30 52(20.2)						
	31–50 48(18.7)						
	>50 16(6.2)						
	**Sex**						
Relapse	Male 15(68.2)	2.16	(0.85–5.47)	0.10			
	Female 7(31.8)	1					
Treatment failure	Male 21(55.3)	1.24	(0.62–2.46)	0.53			
	Female 21(55.3)	1					
Chronic	Male 10(47.6)	0.91	(0.37–2.23)	0.84			
	Female 11(52.4)	1					
Responsive	Male 128(49.8)						
	Female 129(50.2)						
	**Education**						
Relapse	Illiterate 9(40.9)	3.00	(0.78–11.51)	0.10			
	Primary and secondary education 10(45.5)	2.41	(0.64–9.06)	0.19			
	High school and university education 3(13.6)	1					
Treatment failure	Illiterate 16(42.1)	4.00	(1.27–12.51)	0.01			
	Primary and secondary education 18(47.4)	3.25	(1.06–10.01)	0.03			
	High school and university education 4(10.5)	1					
Chronic	Illiterate 10(47.6)	3.33	(0.88–12.58)	0.07			
	Primary and secondary education 8(38.1)	1.93	(0.49–7.51)	0.34			
	High school and university education 3(14.3)	1					
Responsive	Illiterate 76 (29.6)						
	Primary and secondary education 105(40.9)						
	High school and university education 76(29.6)						
	**Occupation**						
Relapse	Employed 15(68.2)	0.41	(0.16–1.08)	0.07	0.26	(0.07–0.91)	0.03
	Unemployed 7(31.8)	1			1		
Treatment failure	Employed 36(94.7)	7.22	(0.96–54.13)	0.05	29.10	(1.79–471.83)	0.01
	Unemployed 2(5.2)	1			1		
Chronic	Employed 18(85.7)	1.17	(0.33–4.15)	0.80	5.98	(0.40–89.42)	0.19
	Unemployed 3(14.3)	1			1		
Responsive	Employed 215(83.7)						
	Unemployed 42(16.3)						
	**Marital status**						
Relapse	Single 15(68.2)	1.07	(0.42–2.74)	0.87	1.42	(0.34–5.79)	0.62
	Married 7(31.8)	1			1		
Treatment failure	Single 32(84.2)	2.68	(1.08–6.66)	0.03	5.34	(1.12–25.35)	0.03
	Married 6(15.8)	1			1		
Chronic	Single 13(61.9)	0.81	(0.32–2.04)	0.66	46.87	(4.68–469.59)	<0.001
	Married 8(38.1)	1			1		
Responsive	Single 171(66.5)						
	Married 86(33.5)						

**Table 3:** Univariate and multiple multinomial logistic regression analyses for clinical risk-related factors: odds ratio of formation of chronic, treatment failure and relapse forms compared to responsive patients with anthroponotic cutaneous leishmaniasis in southeastern Iran.

Treatment outcome	Variables No. (%)	Univariate multinomial logistic regression			Multiple multinomial logistic regression		
		OR	CI (95%)	P-value	OR	CI (95%)	P-value
	**History of chronic diseases** ^a^						
Relapse	Yes 4(18.2)	2.15	(0.67–6.89)	0.19	3.06	(0.64–14.52)	0.15
	No 18(81.8)	1			1		
Treatment failure	Yes 7(18.4)	2.19	(0.87–5.50)	0.09	6.67	(1.37–32.43)	0.01
	No 31(81.6)	1			1		
Chronic	Yes 16(76.2)	31.06	(10.46–92.26)	<0.001	134.78	(82.12–218.39)	<0.001
	No 5(23.8)	1			1		
Responsive	Yes 24(90.7)						
	No 233(9.3)						
	**Course treatment**						
Relapse	Complete ^b^ 16(72.7)	1.47	(0.55–3.96)	0.43			
	Incomplete ^c^ 6(27.3)	1					
Treatment failure	Complete 34(89.5)	0.46	(0.15–1.36)	0.16			
	Incomplete 4(10.5)	1					
Chronic	Complete 13(61.9)	2.42	(0.95–6.16)	0.06			
	Incomplete 8(38.1)	1					
Responsive	Complete 205 (79.8)						
	Incomplete 52(20.2)						
	**Duration of lesion**						
Relapse	>12 1(4.5)	5.58	(0.53–57.90)	0.15	2.69	(0.19–37.46)	0.46
	5–12 15 (68.2)	2.81	(1.05–7.48)	0.03	3.09	(1.06–8.96)	0.03
	<5 6(27.3)	1			1		
Treatment failure	>12 9(23.7)	301.50	(30.44–2985.89)	<0.001	96.56	(7.60–132.37)	<0.001
	5–12 28(73.7)	31.52	(4.22–235.28)	0.001	31.44	(4.06–243.02)	<0.001
	<5 1(2.6)	1			1		
Chronic	>12 9(42.8)	301.5	(30.44–2985.89)	<0.001	100.84	(49.65–202.02)	<0.001
	5–12 11(52.4)	12.38	(1.57–97.37)	0.01	4.19	(0.32–53.48)	0.27
	<5 1(4.8)	1			1		
Responsive	>12 4(1.6)						
	5–12 119(46.3)						
	<5 134(52.1)						
	**Number of lesion**						
Relapse	≤2 18(81.8)	0.87	(0.28–2.72)	0.82			
	>2 4(18.2)	1					
Treatment failure	≤2 34(89.5)	1.66	(0.56–4.92)	0.36			
	>2 4(10.5)	1					
Chronic	≤2 20(95.2)	3.90	(0.51–29.90)	0.18			
	>2 1(4.8)	1					
Responsive	≤2 215(83.7)						
	>2 42(16.3)						
	**Location of lesion**						
Relapse	Hands 5(22.7)	0.67	(0.15–2.91)	0.59	0.76	(0.16–3.62)	0.73
	Face 14(63.6)	3.88	(1.06–14.23)	0.04	5.64	(1.36–23.33)	0.01
	Other 3(13.6)	1			1		
Treatment failure	Hands 13(34.2)	1.31	(0.41–4.20)	0.64	2.68	(0.60–11.86)	0.19
	Face 21(55.3)	4.37	(1.41–13.51)	0.01	6.49	(1.52–27.69)	0.01
	Other 4(10.5)	1			1		
Chronic	Hands 10(47.6)	2.02	(0.42–9.52)	0.37	2.98	(0.26–33.53)	0.37
	Face 9(42.9)	3.75	(0.77–18.08)	0.10	4.10	(0.29–58.08)	0.29
	Other 2(9.5)	1			1		
Responsive	Hands 136(52.9)						
	Face 66(25.7)						
	Other 55(21.4)						

^a^ History of chronic diseases included diabetes, high blood pressure, opium addiction, tuberculosis, allergy, and cardiovascular problems

^b^ Complete: the patients who received a full course of treatment schedule (intramuscular (IM) treatment or intralesional (IL) along with cryotherapy)

^c^ Incomplete: the patients who did not receive a complete course.

**Table 4:** Univariate and multiple multinomial logistic regression analyses for environmental risk related-factors: odds ratio of formation of chronic, treatment failure and relapse forms compared to responsive cases with anthroponotic cutaneous leishmaniasis in southeastern Iran.

Treatment outcome	Variables No. (%)	Univariate multinomial logistic regression			Multiple multinomial logistic regression		
		OR	CI (95%)	P-value	OR	CI (95%)	P-value
	**Building condition**						
Relapse	Unsuitable^a^ 16(72.7)	1.67	(0.63–4.41)	0.30			
	Suitable^b^ 6(27.3)	1					
Treatment failure	Unsuitable 27(71.1)	1.53	(0.73–3.23)	0.25			
	Suitable 11(28.9)	1					
Chronic	Unsuitable 14(66.7)	1.25	(0.48–3.21)	0.63			
	Suitable 7(33.3)	1					
Responsive	Unsuitable 158(61.5)						
	Suitable 99(38.5)						
	**Wall condition**						
Relapse	Unsuitable 18(81.8)	1.71	(0.56–5.25)	0.34			
	Suitable 4(18.2)	1					
Treatment failure	Unsuitable 31(81.6)	1.69	(0.71–4.01)	0.23			
	Suitable 7(18.4)	1					
Chronic	Unsuitable 14(66.7)	0.76	(0.29–1.96)	0.57			
	Suitable 7(33.3)	1					
Responsive	Unsuitable 186(72.4)						
	Suitable 71(27.6)						
	**Door and window net**						
Relapse	No 18(81.8)	1.55	(0.50–4.76)	0.43			
	Yes 4(18.2)	1					
Treatment failure	No 31(81.6)	1.53	(0.64–3.64)	0.33			
	Yes 7(18.4)	1					
Chronic	No 18(85.7)	2.07	(0.59–7.26)	0.25			
	Yes 3(14.3)	1					
Responsive	No 191(74.3)						
	Yes 66(25.7)						
	**Interior housing condition**						
Relapse	Unsuitable 13(59.1)	2.77	(1.14–6.74)	0.02			
	Suitable 9(40.9)	1					
Treatment failure	Unsuitable 17(44.7)	1.55	(0.78–3.09)	0.21			
	Suitable 21(55.3)	1					
Chronic	Unsuitable 9(42.9)	1.44	(0.58–3.54)	0.42			
	Suitable 12(57.1)	1					
Responsive	Unsuitable 88(34.2)						
	Suitable 169(65.8)						
	**Out of building toilet**						
Relapse	Yes 17(77.3)	1.59	(0.56–4.46)	0.37			
	No 5(22.7)	1					
Treatment failure	Yes 26(68.4)	1.01	(0.48–2.11)	0.96			
	No 12(31.6)	1					
Chronic	Yes 18(85.7)	2.81	(0.80–9.81)	0.10			
	No 3(14.3)	1					
Responsive	Yes 175(68.1)						
	No 82(31.9)						
	**Dwelling hygiene condition**						
Relapse	Suitable 6(27.3)	0.45	(0.17–1.20)	0.11			
	Unsuitable 16(72.7)	1					
Treatment failure	Suitable 19 (50)	1.21	(0.61–2.40)	0.57			
	Unsuitable 19 (50)	1					
Chronic	Suitable 5(23.8)	0.38	(0.13–1.06)	0.06			
	Unsuitable 16(76.2)	1					
Responsive	Suitable 116(45.1)						
	Unsuitable 141(54.9)						
	**Number of window**						
Relapse	≥5 13(59.1)	0.95	(0.39–2.30)	0.91			
	<5 9(40.9)	1					
Treatment failure	≥5 29(76.3)	2.12	(0.96–4.66)	0.06			
	<5 9(40.9)	1					
Chronic	≥5 14(66.7)	1.31	(0.51–3.37)	0.56			
	<5 7(33.3)	1					
Responsive	≥5 155(60.3)						
	<5 102(39.7)						
	**Number of room**						
Relapse	<3 8(36.4)	0.71	(0.14–3.61)	0.68			
	3–5 12(54.5)	1.05	(0.22–5.07)	0.94			
	>5 2(9.1)	1					
Treatment failure	<3 12(31.6)	0.71	(0.18–2.76)	0.63			
	3–5 23(60.5)	1.35	(0.37–4.91)	0.64			
	>5 3(7.9)	1					
Chronic	<3 9(42.9)	1.61	(0.19–13.42)	0.65			
	3–5 11(52.4)	1.94	(0.23–15.83)	0.53			
	>5 1(4.8)	1					
Responsive	<3 117(45.5)						
	3–5 119(46.3)						
	>5 21(8.2)						
	**Presence of domestic animal in house**						
Relapse	Yes 11(50)	1.47	(0.61–3.51)	0.38	2.09	(0.79–5.51)	0.13
	No 11(50)	1			1		
Treatment failure	Yes 15(39.5)	0.95	(0.47–1.92)	0.90	1.17	(0.48–2.89)	0.72
	No 23(60.5)	1			1		
Chronic	Yes 13(61.9)	2.39	(0.95–5.97)	0.06	5.20	(1.08–24.98)	0.03
	No 8(38.1)	1			1		
Responsive	Yes 104(40.5)						
	No 153(59.5)						
	**Presence of dog in house**						
Relapse	Yes 4(18.2)	1.28	(0.41–3.99)	0.67			
	No 18(81.8)	1					
Treatment failure	Yes 9(23.7)	1.78	(0.78–4.07)	0.16			
	No 29(76.3)	1					
Chronic	Yes 3(14.3)	0.96	(0.27–3.42)	0.95			
	No 18(85.7)	1					
Responsive	Yes 38(14.8)						
	No 219(85.2)						
	**Solid waste management**						
Relapse	Suitable 21(95.5)	4.70	(0.61–35.81)	0.13			
	Unsuitable 1(4.5)	1					
Treatment failure	Suitable 36(94.7)	4.02	(0.93–17.32)	0.06			
	Unsuitable 2(5.3)	1					
Chronic	Suitable 20 (95.2)	4.47	(0.58–34.18)	0.14			
	Unsuitable 1(4.8)	1					
Responsive	Suitable 210(81.7)						
	Unsuitable 47(18.3)						
	**Presence of dog in region**						
Relapse	Yes 18(81.8)	1.06	(0.34–3.27)	0.91			
	No 4(18.2)	1					
Treatment failure	Yes 35(92.1)	2.74	(0.81–9.30)	0.10			
	No 3(7.9)	1					
Chronic	Yes 20(95.2)	4.71	(0.61–35.95)	0.13			
	No 1(4.8)	1					
Responsive	Yes 208(80.9)						
	No 49(19.1)						

^a^ Unsuitable: denotes the patients who live in inappropriate housing conditions in terms of building, walls, interior housing with no cracks and crevices and also solid waste management and dwelling hygiene condition

^b^ Suitable: denotes the patients who live in an appropriate housing condition in terms of building, walls, interior housing with no cracks and crevices and also appropriate solid waste management and dwelling hygienic condition.

Based on the univariate and multiple multinomial logistic regression analyses, 8 major determinants among 23 risk-related factors including nationality, age group, occupation, marital status, history of underlying chronic diseases, duration of the lesion, location of lesion and presence of a domestic animal in the house were significantly related with the formation of non-healing forms (relapse, treatment failure and chronic) in southeastern Iran.

### Demographical factors

The odds of development of chronic forms (OR = 8.10, CI = 1.43–45.79, P = 0.01) or treatment failure forms (OR = 3.41, CI = 1.11–10.43, P = 0.03) than the responsive forms in Afghani patients were significantly higher than the Iranian cases. Treatment failure cases than the responsive forms in patients aged <8 years (OR = 0.19, CI = 0.03–0.97, P = 0.04) or aged 8–15 years (OR = 0.09, CI = 0.01–0.44, P = 0.003) displayed significantly higher odds than those with other age groups.

The odds of creating relapse forms than responsive forms in employed patients (OR = 0.26, CI = 0.07–0.91, P = 0.03) than unemployed patients or odds of treatment failure forms than responsive forms in employed patients (OR = 29.10, CI = 1.79–471.83, P = 0.01) than unemployed patients were significantly different. Also, the development of chronic forms (OR = 46.87, CI = 4.68–469.59, P<0.001) or treatment failure forms (OR = 5.34, CI = 1.12–25.35, P = 0.03) compared to responsive forms in single patients were significantly higher than those married cases.

### Clinical factors

The odds of developing chronic forms (OR = 134.78, CI = 82.12–218.39, P<0.001) or treatment failure forms (OR = 6.67, CI = 1.37–32.43, P = 0.01) relative to responsive forms in patients with a history of underlying chronic diseases were significantly higher than cases without a history of underlying chronic diseases. Formation of chronic forms compared to responsive forms in cases referred >12 months following the onset of the lesion (OR = 100.84, CI = 49.65–202.02, P<0.001) was significantly higher than those <5 months duration of the cutaneous lesion. The odds of development of treatment failure forms than responsive forms in cases referred >12 months following the onset of the lesion (OR = 96.56, CI = 7.60–132.37, P<0.001) or 5 to 12 months old (OR = 31.44, CI = 4.06–243.02, P<0.001) were significantly higher than those referred <5 months duration of the lesion. The odds of relapse forms than responsive forms in cases referred 5 to 12 months following the onset of the lesion (OR = 3.09, CI = 1.06–8.96, P = 0.03) had significantly higher odds than those <5 months duration of the cutaneous lesion. Moreover, the odds of relapse development (OR = 5.64, CI = 1.36–23.33, P = 0.01) or treatment failure forms (OR = 6.49, CI = 1.52–27.69, P = 0.01) than responsive forms in patients with facial lesion were significantly higher than other anatomical locations.

### Environmental factors

The odds of developing chronic forms (OR = 5.20, CI = 1.08–24.98, P = 0.03) than the responsive form in patients keeping domestic animals in the house were significantly higher than cases not having domestic animals in the house.

### Other factors

Other demographical, clinical and environmental factors including sex, education, course of treatment, number of lesions, building, interior housing and wall conditions, dwelling hygienic condition, door and window nets, out of building a toilet, number of window and room, presence of a dog in the house and surrounding areas and solid waste management were not significantly associated with the formation of chronic, treatment failure or relapse cases relative to the responsive group.

## Discussion

In Iran, CL is one of the major health complications, particularly in Kerman province, southeast of Iran[21, 32]. The majority of the patients infected in Kerman district were ACL due to *L*. *tropica*[14]. However, in this study, the effort was made to using molecular techniques for the characterization of the *Leishmania* isolates. Based on the nested PCR technique all isolates were identified to be *L*. *tropica* species in the present study.

Unresponsiveness to treatment may be occurred by host factors, treatment compositions, and parasite features [3, 13, 32, 33]. Resistance to drugs is also a serious problem for the treatment of many diseases such as leishmaniasis[13, 34, 35].

Based on previously published papers, histopathological findings of various clinical forms of ACL with variable morphological changes were represented [36, 37].

Our histopathological findings showed that in responsive form, histiocytes were filled with many and/or with few intracytoplasmic Leishman bodies, while in other forms (relapse, chronic and treatment failure forms), Leishman bodies were not present or rarely could be found. Moreover, the histopathological findings were different from one form to another form, and these differences were quite comparable as mentioned in the results[36]. Also, immunohistochemical findings showed a decrease in the number of histiocytes, an increased number of lymphocytes and fibrosis in responsive forms than unresponsive ones (relapse, treatment failure and chronic forms) [38–41]. The progress of the disease in response to infection can be severely affected by the pathogen burden and immunopathology caused by the infection [42, 43]. The histopathological and immunohistochemical findings will be helpful to advance our understanding about the disease and its clinical forms.

The current study showed that CD1a epidermal, dermal DCs, and CD68 macrophages are the most important cells that regulate the outcome of leishmaniasis. In our immunohistochemical findings, all mentioned cells were prominent in the responsive form. In contrast, whilst in the chronic and treatment failure forms, the density of CD68 cells was minimal; CD20 cells were notable. Following the *Leishmania* infection, the infiltration of CD68 macrophages and CD1a DCs phagocytose Leishman bodies leads to the production of IL-12 and, in turn, the generation of IFN-γ and promotion of the parasite death [37, 44].

Moreover, the results of this study showed that the development of chronic (P< 0.01) or treatment failure forms (P = 0.03) in Afghani patients was significantly higher than those in Iranian patients. Afghani people due to their social lifestyle provide their initial essentials in difficulty and also are more frequently exposed to CL and other diseases and often receive their medications too late and in an irregular manner. Therefore, the possibility of developing unresponsive forms increases in this group. One of the main factors that can be effective in poor treatment adherence (poor compliance to treatment for any reason) is poverty and social-economic factors [13, 45].

This study revealed that the formation of treatment failure in cases with ACL was significantly associated with lower age in <8 years old (P = 0.04) or 8–15 years old (P = 0.03) groups. Probably, deficiencies in cell immune response caused by several debilitating factors such as immature immune systems play a leading role in the promotion of treatment failure forms in children. It is well known that the control of CL is closely associated with the cellular immune system. Formerly, reports showed that the ACL incidence rate of lupoid leishmaniasis (leishmaniasis recidivans cutis) in the endemic foci of Kerman in children was 18.7% compared to 4.7% of other age groups[46]. One of the major risk factors is poor treatment adherence which could be a serious public health threat associated with the development of resistance to drugs. Usually, children receive suboptimal treatment schedule to the pain induced by parental administration and poor compliance. Additionally, the low-dose drug, poor cooperation and partial adherence during the course of receiving the medication could be other factors that contribute to such a treatment outcome. This behavioral interference in receiving a partial treatment regimen frequently occurs in children.

The results also showed that the development of chronic (P<0.001) or treatment failure (P = 0.03) forms in single patients were significantly higher than those in married ones. These findings are consistent with those found by other studies which demonstrated that married individuals represented a suitable immune function relative to the comparable divorced ones [47]. Many studies have focused particularity on depressive signs, which are lesser among married individuals than unmarried ones[48].

Social stigma, disfigurement, and depression are some of the most identifiable features of neglected tropical diseases particularly of patients with CL[49]. Moreover, as the results, depression disorders can sensitize the inflammation, consequently stimulating larger cytokines to increase in response to pathogen and stressor factors [50]. Both depression and stress increase the risk of proliferation of infectious agents, prolonging infections and hence enhancing the development of non-healing forms [51, 52].

The results also showed that the generation of relapse in patients with ACL who had an occupation was significantly lower than those of jobless cases. The causes of this finding are not well understood. Presumably, deficiency in cellular immune response due to several factors such as stress caused by more worry to survive the lesion scar after healing (because these individuals have more free time in the home and more think about their lesion) plays a role in the promotion of relapse forms in unemployed people. Also, the findings showed that the treatment failure (P = 0.01) form in patients that had an occupation was significantly more than those who did not have an occupation. As mentioned before because CL is a poverty-stricken disease and is often prevalent in poor people. Also if they start to take medications it is usually incomplete, irregular and with long duration of treatment (poor treatment adherence). The drug resistance of the *Leishmania* species is frequently due to low adherence to treatment regimen [13, 24, 53].

In patients with underlying chronic diseases, the increased incidence of infection is affected by the immune system. For example, patients with diabetes mellitus (DM) have more chances of acquiring infections compared with the ones lacking DM. Hence, the increased prevalence of infections can be intensified by a disorder and weakness in the immune system[34, 35]. It is worth mentioning that addiction of opium is a serious health problem in the CL endemic areas in the southeast of Iran and adjacent countries[30]. A study in 2014 revealed that CL lesions in opium-addicted people were more severe compared to control cases in terms of the number, size, and duration of the disease[30], although the actual reason is not well clear. In this study, patients with the underlying chronic diseases like diabetes, high blood pressure, opium addiction, tuberculosis, allergy, and cardiovascular problems had more significantly developed chronic (P<0.001) or treatment failure (P = 0.01) forms than those lacking a history of chronic diseases. This finding revealed that a history of chronic diseases was also identified to be a risk factor for the development of the chronic and treatment failure forms in cases with ACL. It seems that among the risk associated-factors, a history of chronic diseases with a significant odds ratio plays a critical role in the treatment outcome.

In the present study, patients who started the treatment after >12 months, had higher odds in formation of chronic or treatment failure forms (P<0.001) than those who commenced treatment less than 5 months. Also, patients who started the treatment after 5–12 months, represented higher odds in the creation of relapse (P = 0.03) or treatment failure forms (P<0.001) than the ones who had started treatment less than 5 months. In the absence of active-case findings and lack of knowledge among the people, arbitrary therapy, and unsuitable health care system, the disease was late detected. Hence, in this group of patients, the conventional course of treatment has been more problematic. Incomplete or irregular and eventually poor treatment of CL sometimes caused the promotion of drug-resistant variants to *Leishmania* [24, 53].

The findings herein demonstrated that the development of significant cases of relapse and treatment failure forms in those who had lesions on the face was significantly higher than those with lesions on hands and other parts of the body. The precise reasons for the presence of facial relapse and treatment failure are not well known. According to our results, most of the lupoid lesions (relapse) happened on the face. This clinical syndrome is an abundant sequel of ACL due to *L*. *tropica* in the Old World such as Iran[9, 54, 55]. The majority of the lupoid lesions are highly resistant to MA[40, 56]. Several factors have been involved as the basis of relapse and treatment failure such as deficient immune response and poor treatment adherence [9, 21].

The results also showed that the development of chronic form in patients having domestic animals was significantly higher than those not having domestic animals (P = 0.03). The rate of sand flies in the houses that having domestic animals is significantly higher than those who do not have one [57, 58]. The individuals who live in these houses in endemic areas are more prone to vectors’ bite and parasite infection due to suitable conditions for propagation[57, 58]. In such circumstances, there is a greater chance of re-infection and induction of multiple lesions caused by more density of vectors. Hence, it seems that the re-infection and multiple lesions are increased because of the sand fly density and parasite load which enhances the chance of developing chronic form. In this regards, various genotypes within the same species of *Leishmania* may respond differently to drugs because some intraspecific genetic heterogeneities have previously been reported for *L*. *tropica* from similar region[14, 59, 60]. Furthermore, chronic form in patients with multiple lesions can be produced by a poor immune response against the *Leishmania* species[1, 21].

In general, protecting individuals from being bitten by sandflies is a vital and important measure for the prevention of refractory forms of ACL caused by *L*. *tropica* in southeastern Iran. Since human is the principal reservoir host, early detection, prompt diagnosis, effective and timely treatment could play a fundamental role in the control of the disease. Knowledge of the major risk factors for ACL infection is essential in improving clinical and public health strategies.

The main strength of the current study was the well-equipped Health Clinic associated with the Leishmaniasis Research Center, strong registry systems, and robust infrastructures that manage the patients by expert staff, trained physicians, and the well-organized team works with appropriate facilities for diagnosis, treatment and follow up assessments. On the other hand, however, this study had a major limitation that was although initially, a larger number of unresponsive cases participated voluntarily in the study as time passed they were lost or absent to be visited. It is of note to mention that as the largest province, Kerman is a vast and diverse complex and possesses many rural communities located in remote areas.

In conclusion, 8 major risk factors, including nationality, age groups, occupation, marital status, history of chronic diseases, duration of the lesion, the lesion on face and presence of domestic animals in the house were significantly linked with the induction of unresponsive forms such as chronic, treatment failure and relapse. Poor treatment adherence has a strong negative impact on treatment outcomes. Regular monitoring of unresponsiveness to drugs and recognition of leading factors linked with chronic, treatment failure and relapse forms of ACL is crucial for proper prophylactic and therapeutic strategic plans. The present findings clearly showed a positive association between ACL and distinct demographic, clinical and environmental risk determinants. Also, the histopathological and immunohistochemical findings will be helpful to improve our knowlege about the several clinical forms of ACL and its diverse histophatogical changes. Therefore, to overcome this serious public health problem, clinical practitioners and health surveillance staff should be aware of and monitor such perplexing factors to be able to achieve a comprehensive control program and treatment strategy.

## Supporting information

S1 STROBE checklist (DOCX)
